# Supplementary material for: An acoustic-based method for locating maternity colonies of rare woodland bats
Source: PeerJ. 2023 Oct 3;11:e15951. doi: 10.7717/peerj.15951 (PMC10557938; doi:10.7717/peerj.15951)
Supplement: Supplemental Information 1 [file peerj-11-15951-s001.pdf]

Sampling rate: 192 kHz; trigger: trig level = 18 SNR, trig window = 2.0 seconds; gain: SMX-U1 = 12 dB, SMX-US = 48 dB; high-pass filter: HPF left = 4 kHz; and low-pass filter: LPF left = OFF. Sampling occurred from 30 minutes prior to sunset until 30 minutes after sunrise for a minimum of three consecutive detector-nights at each sampling point.
