# Supplementary material for: An acoustic-based method for locating maternity colonies of rare woodland bats
Source: PeerJ. 2023 Oct 3;11:e15951. doi: 10.7717/peerj.15951 (PMC10557938; doi:10.7717/peerj.15951)
Supplement: Supplemental Information 2 [file peerj-11-15951-s002.pdf]

Weather stations did not record any rainfall during survey periods, or sunset temperatures below 11.6 °C (mean = 16.6 °C). In addition, average ground level wind speeds did not exceed 2.3 m s^{-1}, substantially lower than wind speeds (11 m s^{-1}) reported by Davidson-Watts (2014, unpublished data), in which radio-tagged barbastelles continued to display the same behaviour as on calmer nights.
