# Supplementary material for: An acoustic-based method for locating maternity colonies of rare woodland bats
Source: PeerJ. 2023 Oct 3;11:e15951. doi: 10.7717/peerj.15951 (PMC10557938; doi:10.7717/peerj.15951)
Supplement: Supplemental Information 3 [file peerj-11-15951-s003.pdf]

In general, the value of alpha chosen depends on the purpose of the hull, as well as the quality, quantity, and spread of the data. Here, we selected a threshold of 0.7 by increasing the value of alpha incrementally for each set of points; to represent the best balance between the inclusion of all points within each site and the exclusion of areas in which we did not deploy detectors. We added an additional buffer to the final hull polygons to provide smoothing to the hull and to ensure that we incorporated woodland edges into the hull where appropriate. We determined the size of the buffer by calculating the overall median nearest neighbour distance between any two given detectors. Subsequently, we overlaid the woodland layers and clipped them to the buffer.
