# Supplementary figures and images for: An acoustic-based method for locating maternity colonies of rare woodland bats
Source: PeerJ. 2023 Oct 3;11:e15951. doi: 10.7717/peerj.15951 (PMC10557938; doi:10.7717/peerj.15951)

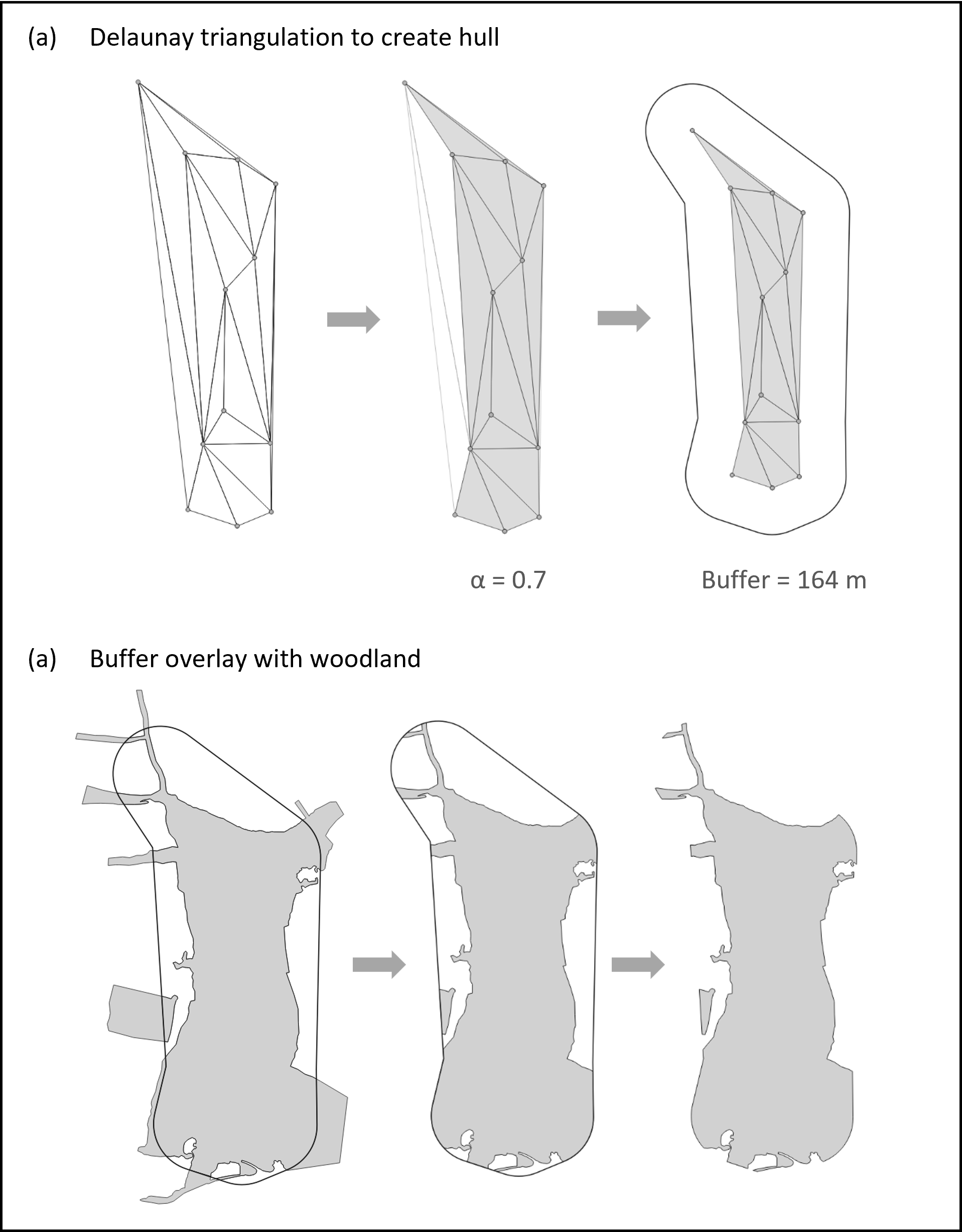

Supplement: Supplemental Information 4 — (A) A hull that encompasses all detector locations is created and a buffer added to the hull. The grey lines show the Delaunay triangulation which is the starting point of the alpha hull algorithm. Points represent acoustic detector locations. (B) The buffer is overlaid with the woodland layer and the woodland clipped to the buffer. [file peerj-11-15951-s004.png]

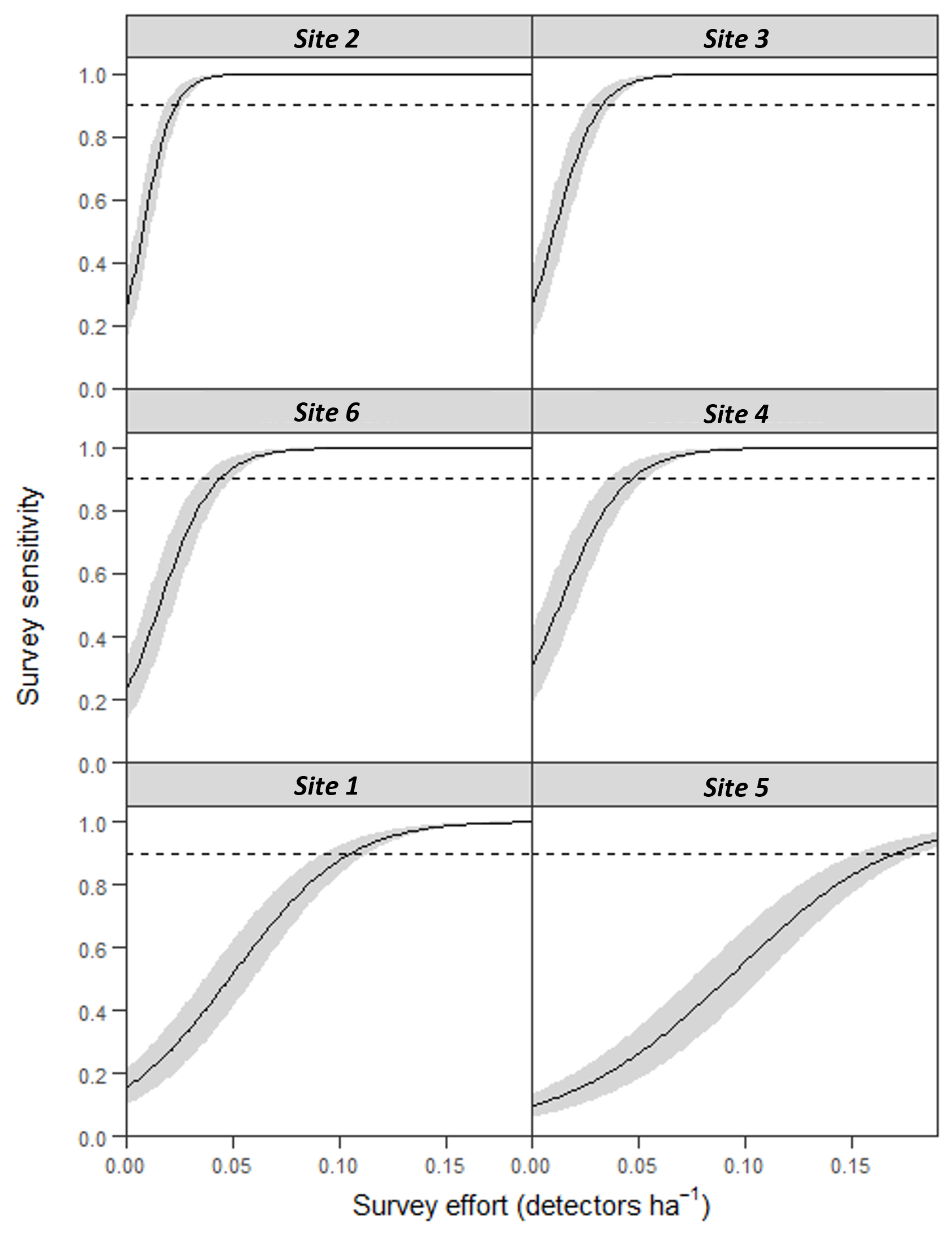

Supplement: Supplemental Information 5 — Graphs show predictions (solid line) from a generalised additive mixed model (GAMM), with 95% confidence limits (grey area). Survey sensitivity represents the probability of correctly identifying a woodland with a colony, calculated for various detector densities. It is estimated as the proportion of 1,000 stochastic simulations in which at least one static detector in a woodland exceeds the optimal cut-off threshold of 3.45 bat passes (within one hour of sunset). Dashed line indicates the desired survey sensitivity of 0.90. [file peerj-11-15951-s005.png]

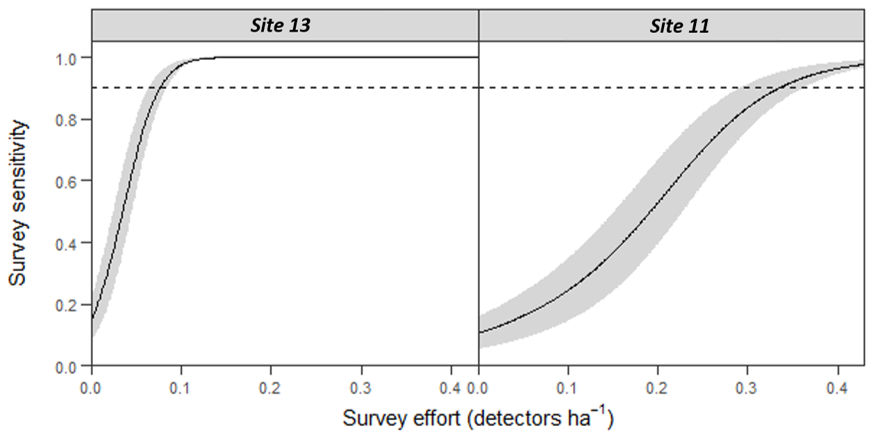

Supplement: Supplemental Information 6 — Graphs show predictions (solid line) from a generalised additive mixed model (GAMM), with 95% confidence limits (grey area). Survey sensitivity represents the probability of correctly identifying a woodland with a colony, calculated for various detector densities. It is estimated as the proportion of 1,000 stochastic simulations in which at least one static detector in a woodland exceeds the optimal cut-off threshold of 3.45 bat passes (within one hour of sunset). Dashed line indicates the desired survey sensitivity of 0.90. [file peerj-11-15951-s006.png]
