# Supplementary material for: An acoustic-based method for locating maternity colonies of rare woodland bats
Source: PeerJ. 2023 Oct 3;11:e15951. doi: 10.7717/peerj.15951 (PMC10557938; doi:10.7717/peerj.15951)
Supplement: Supplemental Information 7 [file peerj-11-15951-s007.docx]

| Passes | Probability |
| --- | --- |
| 0 | 0.03 |
| 1 | 0.10 |
| 2 | 0.25 |
| 3 | 0.53 |
| 4 | 0.78 |
| 5 | 0.92 |
